# Transmission of scrapie prions to primate after an extended silent incubation period

**DOI:** 10.1038/srep11573

**Published:** 2015-06-30

**Authors:** Emmanuel E. Comoy, Jacqueline Mikol, Sophie Luccantoni-Freire, Evelyne Correia, Nathalie Lescoutra-Etchegaray, Valérie Durand, Capucine Dehen, Olivier Andreoletti, Cristina Casalone, Juergen A. Richt, Justin J. Greenlee, Thierry Baron, Sylvie L. Benestad, Paul Brown, Jean-Philippe Deslys

**Affiliations:** 1 Atomic Energy Commission (CEA), Institute of Emerging Diseases and Innovative therapies (iMETI), Division of Prions and related Diseases (SEPIA), 18 Route du Panorama, BP6, 92265 Fontenay-aux-Roses, France; 2 INRA-ENVT 23 Chemin des Capelles, 31076 Toulouse, France; 3 Istituto Zooprofilattico Sperimentale del Piemonte, Via Bologna 148, 10154 Torino, Italy; 4 Virus and Prion Research Unit, National Animal Disease Center, USDA, Agricultural Research Service, 1920 Dayton Avenue, 50010 Ames, Iowa, USA; 5 ANSES, 59 Rue Tony Garnier, 69364 Lyon, France; 6 Norwegian Veterinary Institute, Ullevalvelen 68, 0106 Oslo, Norway

## Abstract

Classical bovine spongiform encephalopathy (c-BSE) is the only animal prion disease reputed to be zoonotic, causing variant Creutzfeldt-Jakob disease (vCJD) in humans and having guided protective measures for animal and human health against animal prion diseases. Recently, partial transmissions to humanized mice showed that the zoonotic potential of scrapie might be similar to c-BSE. We here report the direct transmission of a natural classical scrapie isolate to cynomolgus macaque, a highly relevant model for human prion diseases, after a 10-year silent incubation period, with features similar to those reported for human cases of sporadic CJD. Scrapie is thus actually transmissible to primates with incubation periods compatible with their life expectancy, although fourfold longer than BSE. Long-term experimental transmission studies are necessary to better assess the zoonotic potential of other prion diseases with high prevalence, notably Chronic Wasting Disease of deer and elk and atypical/Nor98 scrapie.

For several protein-misfolding neurodegenerative diseases (Alzheimer’s disease, Parkinson’s disease, prion diseases, amyotrophic lateral sclerosis, etc.), a minority of cases are linked to mutations of specific proteins (familial cases, approximately 10%) but the great majority are considered to be sporadic and idiopathic, i.e. without identified origin. Among these proteinopathies, prion diseases constitute a peculiar group as they naturally occur in both humans and animals and are transmissible through natural routes of exposure. In humans, Kuru was transmitted through cannibalistic funeral rites^1^ and Creutzfeldt-Jakob disease (CJD) has been transmitted iatrogenically from the use of growth hormone, grafts, or surgical instruments contaminated with tissue from unidentified human sporadic CJD cases^2^. Sporadic CJD (sCJD) represents more than 80% of human cases of prion disease and is readily transmissible to humans and non-human primates.

In animals, classical scrapie (c-scrapie) of sheep and goats and chronic wasting disease (CWD) in farmed and wild cervids are transmitted through direct contact between animals and through grazing in prion-contaminated environments, which leads to persistence of these diseases at an enzootic level. In contrast, the bovine spongiform encephalopathy (BSE, hereafter called classical BSE, or c-BSE) epizootic was maintained through feeding cattle with prion-contaminated meat and bone meals.

Animal prion diseases were considered to be innocuous for humans until the diagnosis of the first cases of variant CJD in young British patients^3^, which were linked to the consumption of beef products derived from c-BSE-infected cattle. This interspecies transmission was first hypothesized on the basis of epidemiological evidence (the occurrence of all the first human vCJD cases in the country hosting more than 99% of the cattle c-BSE cases) and later confirmed by experimental transmissions in animal models (macaque^4^ and mice^5^). Several strict control measures for c-BSE were successfully adopted in Europe to protect human and animal health during past decades, and were extended to other animal prion diseases such as scrapie. Due to a decreasing prevalence of c-BSE to a point where only rare cases are reported, control measures are being progressively challenged and relaxed.

Prior to the recognition of c-BSE, c-scrapie and sCJD were the main reported prion diseases. The long-held belief that scrapie does not transmit to humans has been based on the lack of significant spatial correlation between c-scrapie and sCJD clusters^6^,^7^ and the fact that Australia and New Zealand, which are nearly free from c-scrapie, have a similar prevalence of sCJD as countries with scrapie cases. On the basis of these epidemiological considerations, c-scrapie was considered unrelated to sCJD and harmless to humans. The emergence of c-BSE dramatically increased the number of studies in the prion field: new prion diseases were identified in both animals and humans, and improved molecular and lesional discrimination approaches provided evidence that several different prion strains or phenotypes could be grouped under the terminologies of “classical scrapie” or “sporadic CJD”. Therefore, it appears obvious that global epidemiological studies devoid of fine discrimination between those different animal and human prion strains would be unable to highlight and identify if a particular animal prion strain could have a potential zoonotic effect.

Historical transmission studies of scrapie to primates have shown variable results^1^,^8^,^9^,^10^, leading to the generally accepted notion that, as of yet, there was no clear experimental evidence that scrapie prions are associated with prion disease in humans^11^, even if the species barrier from humans to small ruminants can be crossed since sCJD induces a scrapie-like prion disease in goats^12^. Very recently, several scrapie isolates have been shown to induce prion diseases with phenotypic features similar to sporadic CJD in transgenic mice overexpressing human PrP^13^. This recent study provides clear evidence that scrapie has at least a zoonotic potential, even though transmissions occurred at a low rate after a second passage in this rodent model considered as highly susceptible based on the overexpression of human PrP. These observations question the real level of permeability of the human species barrier with regards to scrapie transmission in physiological conditions of PrP expression and the true level of the zoonotic potential of the different scrapie agents.

Transgenic mouse models of human prion diseases complement studies performed in different species of non-human primates, the latter model providing the first evidence of human prion disease transmission^1^,^4^,^9^. Among old-world primates, which are closest to humans in terms of phylogeny, the cynomolgus macaque is the preferred current primate model for the study of human prion diseases^4^,^14^,^15^,^16^. Interestingly, the recent mathematical modeling of vCJD infection based on this model fits well with the current human epidemiology of vCJD^17^,^18^.

We, and others, have used the macaque model to assess the zoonotic potential of other ruminant prion strains in comparison to c-BSE. The more recently identified atypical L-type BSE strain was also transmissible to macaques^19^,^20^ even apparently more efficiently than c-BSE, corroborating other experimental models^21^,^22^.

The successful transmission of two bovine prion strains into macaques after relatively short incubation periods was suggestive of a low cattle-to-primate species barrier and we have thus continued to explore the zoonotic potential of cattle-derived prion isolates (including CWD after passage through cattle). As controls, we used two sheep prion isolates (one c-scrapie and one atypical/Nor98 scrapie) both reputed to be non-transmissible and devoid of risk to humans. We report here that, surprisingly, the isolate of sheep c-scrapie led to clinical prion disease in a cynomolgus macaque a decade after exposure (two to four times longer than the incubation period observed with bovine prion strains) indicating the need for continued research in this area to clarify any potential risks of scrapie to human health and implying that very long term experiments are necessary to properly assess this risk.

## Results

We inoculated cynomolgus macaques through the intracerebral route with different inocula of animal prion diseases. For some of them, inoculation in tonsils also was performed to model oral exposure. The survival periods are shown in table I and compared to the results of c-BSE and L-type BSE experiments previously described by our and other groups, including oral exposures^14^,^16^,^19^,^20^,^23^,^24^. C-BSE inoculated macaques developed disease from 2 to 8 years (93 months) post-inoculation, depending on the dose (25 mg to 50 μg of brain for the lowest doses which transmitted the disease). No further prion disease was observed within c-BSE inoculated macaques for the following four years (ongoing study, 12 years of surveillance). However, one cynomolgus macaque exhibited obvious neurological signs more than 9 years (110 months) after intracerebral exposure to a high dose of a sheep classical scrapie isolate (25 mg of brain). The animal presented with an anxious/nervous disposition, hyperesthesia, and a stiff gait. Trembling of the forelegs when catching objects or food progressively worsened. Weakness was first noted in the right leg and then progressed to both legs together with ataxia and general weakness. After 8 months of clinical progression, the animal was euthanized 118 months post-inoculation.

**Figure 1:**
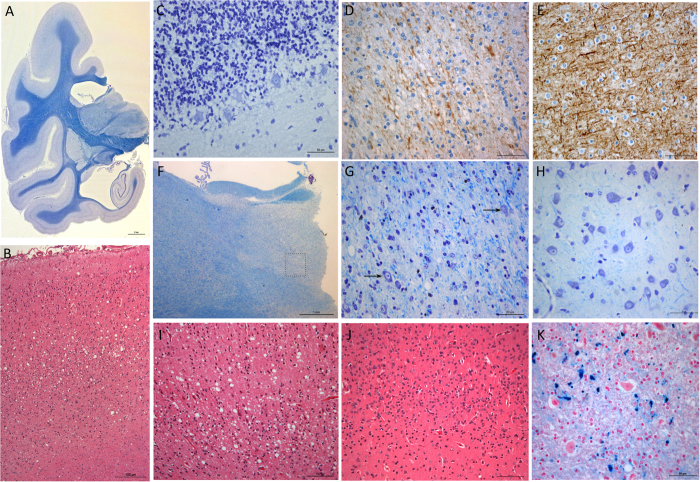
Neuropathological features of scrapie-infected cynomolgus macaque. **A**) Macroscopic view of brain atrophy predominant in temporal lobe and enlarged ventricles, associated with a half-turn rotation of the hippocampal formation (x 1.4, Klüver-Barrera (KB) stain). (**B**) Laminar spongiform change involving mostly layer IV of occipital cortex (x 100 original magnification, Hematoxylin-Eosin (HE)). (**C**) Spongiform change in Purkinje cells of the cerebellar cortex, most of them being shrunken or absent (x 400, KB). (**D**) Axonal loss of the temporal white matter, visualized with monoclonal anti-neurofilament antibody 2F11 (x 400, 2F11 antibody), in comparison to (**E**) a normal neurofilament staining in temporal white matter of a healthy primate (x 400). (**F**) Massive lesion of the dorso-medialis thalamic nucleus (x 25, KB) (**G**) Higher magnification of the thalamic nucleus (corresponding to the inset in panel (**F**) showing massive rarefaction of neurons (arrows marked the few remaining neurons), an increase of the density of glia and spongiosis (x 400, KB), in comparison to (**H**) the same thalamic region in healthy primate. (**I**) Higher magnification of the laminar spongiosis of occipital cortex (x200, HE), in comparison to (**J**) the occipital cortex of healthy primate (x200, HE). (**K**) Iron deposits in the globus pallidus (x 400, Perls).

**Figure 2:**
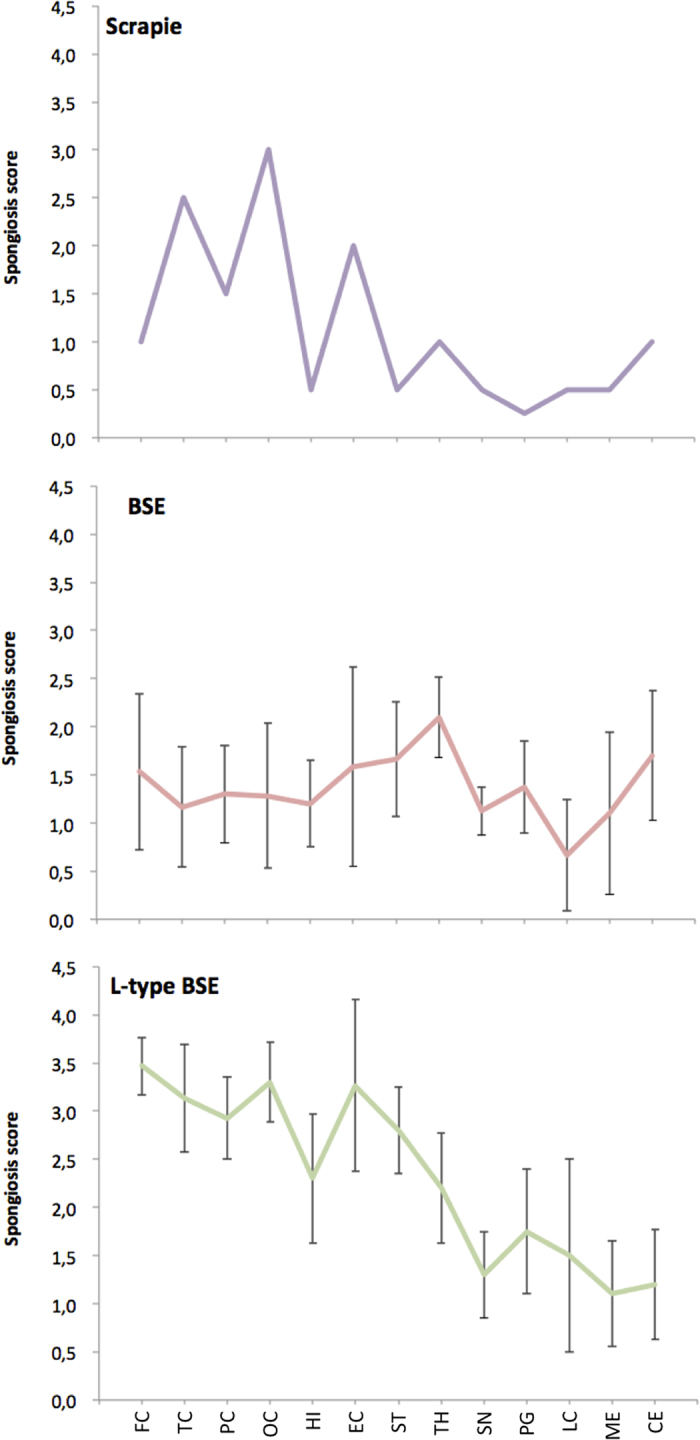
Spongiform lesion profiles in primates infected with various TSEs. Lesion profiles in primates exposed to scrapie (**A**) BSE (**B**) or L-type BSE (**C**) were defined according to the scoring and areas described by Parchi *et al*.^29^. The lesion profile of c-BSE- and L-type BSE-infected primates is depicted as the mean among 5 and 4 primates exposed to c-BSE or L-type BSE, respectively.

At postmortem examination, the brain was atrophic, most evident in the temporal lobes (Fig. 1A). Microscopic examination revealed widespread spongiform change throughout the nervous system, with a distribution of spongiform lesions different from those observed for c-BSE- or L-type BSE-infected macaques (Fig. 2). Lesions were irregularly laminar in the neocortex and more intense in the cingular and occipital lobes (Fig. 1B and 1I compared to 1J), moderate in striatum, thalamus, hippocampus and brain stem with a dorsal predominance, and very mild in the spinal cord except at the lumbar level. There was no vacuolation in lateral cuneate nucleus or the nucleus of the vagus nerve. Conversely, in the cerebellum, spongiosis of the Purkinje cells was so intense that most of them were destroyed (Fig. 1C). Elsewhere, neuronal loss was moderate, increasing from frontal to occipital cortex. Mild neuronal loss also was present in the basal ganglia and the amygdala. Shrunken neurons were noted in entorhinal cortex, subiculum, field CA2 of the hippocampus associated with an axonal loss in temporal lobe (Fig. 1D,E), substantia nigra and locus coeruleus. Optic tracts were practically normal. No long tract degeneration was observed.

A peculiar lesion was present in the medial nucleus of the thalamus including a massive loss of parenchymal elements associated with spongiform change and gliosis (Fig. 1F & 1G vs 1H) but devoid of necrosis or evidence of a vascular abnormality. The mammillary body had spongiform change, but no obvious neuronal loss was observed and the width of the corpus callosum was within normal limits.

Gliosis was present in the hemispheric white matter, the *pars lateralis* of the globus pallidus, the brain stem and the lower spinal cord. Reactive astrocytosis was especially intense in the cerebellum. Iron deposits were noted in the lenticular nucleus, predominant in globus pallidus (Fig. 1K).

**Figure 3:**
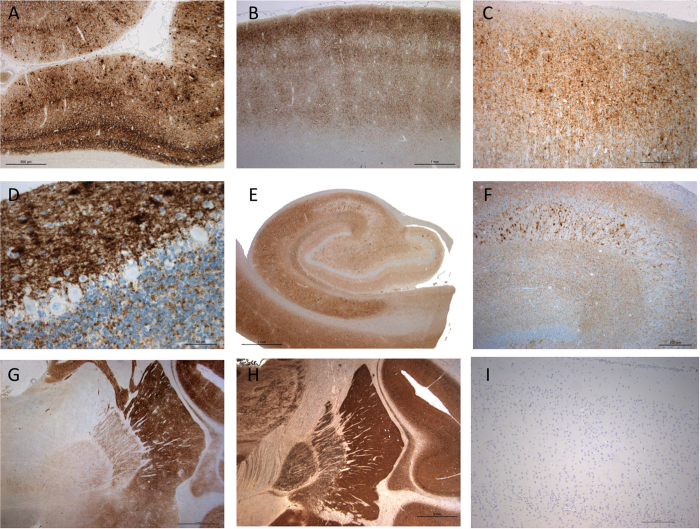
PrP^sc^ immunostaining of CNS of scrapie-infected cynomolgus macaque. **A**) Massive laminar PrP^Sc^ immunoreactivity in layers IV and VI of occipital cortex with many aggregates after PK treatment (x 50- 3F4 monoclonal antibody). (**B**) Predominance of PrP^Sc^ in the superficial layers of the frontal cortex, on top of underlying columns (x 25- 3F4). (**C**) Detail of PrP^sc^ immunoreactivity in the frontal cortex (x100- 3F4). (**D**) PrP^Sc^ synaptic deposits and aggregates in molecular and granule cell layers of cerebellar cortex (x 400- 3F4). (**E**) Ammon’s horn immunostaining showing deposits of PrP^Sc^ in pyramidal cells and abundant aggregates in field CA2 (x 25- 12F10 monoclonal antibody). (**F**) Detail of the aggregates in field CA2 (x100 – 3F4) (**G**) PrP^Sc^ immunostaining marked in striatum while mild in globus pallidus (x 12.5-3F4). (**H**) Immunostaining with polyclonal cytochrome c oxidase antibody with strong immunoreactivity within the neuropil of the putamen (x12.5 -gift of Dr A.Lombes). (**I**) Absence of PrP^C^ staining in the occipital cortex of an healthy control (x100 – 3F4).

PrP^Sc^ immunoreactivity was heavily distributed in cortex, striatum and cerebellum, consisting of synaptic deposits and small and large aggregates (Fig. 3A,B,C,D), whereas PrP^C^ was undetected with this technique (supplementary figure 1). Deposits were predominant in the cingular and occipital cortices, the basal ganglia, and the molecular layer of the cerebellum, and mild in the brainstem and the spinal cord. In the field CA2 of the hippocampus, large aggregates were correlated with neuronal loss (Figs 3E,F). PrP^Sc^ immunoreactivity was also present in the plexiform layers of the retina (data not shown). According to the cerebral atrophy, Alzheimer’s markers were also investigated but were negative (data not shown).

**Figure 4:**
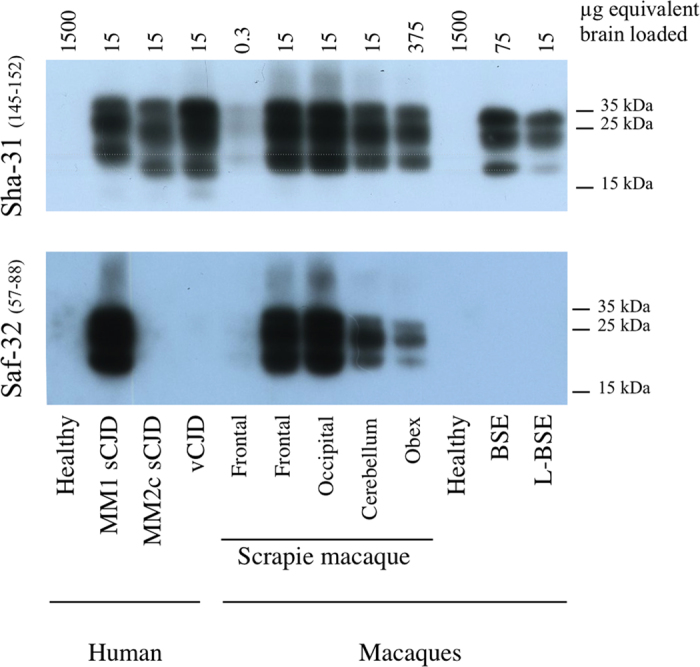
PrP^res^ electrophoretic profile in scrapie-infected macaque. Samples issued from different brain regions of the scrapie-infected macaque (frontal, occipital, cerebellum or obex) were homogenized (20% weight/volume in 5% glucose solution) and then purified according to the TeSeE purification protocol using 4 μg of proteinase K / mg of brain^19^. As controls, samples derived from occipital cortices of patients (healthy, MM1 or MM2c sporadic CJD, variant of CJD) or classical BSE- or L-type BSE-infected macaques were equally treated. Resulting purified PrP^res^ was solubilized in loading buffer, the equivalent of 0.3 to 1,500 μg of brain was loaded on a 12% acrylamide gel (depending on the levels of positivity of each sample), and revealed using anti-PrP monoclonal antibodies targeted against the core (Sha-31 recognizing epitope 145–152) or the octapeptides (SAF-32, recognizing octarepeats included in region 57–88) of the protein.

Western blot analyses confirmed that there were lower amounts of PrP^res^ in brainstem in comparison to the cerebral cortices or cerebellum (Fig. 4). The molecular profile of all western blot samples exhibited PrP^res^ with a type 1 profile characterized by a 21 kDa unglycosylated band revealed with Sha-31 anti-PrP antibody, whereas the samples derived from c-BSE- and L-type BSE-infected primates exhibited PrP^res^ with a type 2 profile (19 kDa) as expected. Moreover, the N-terminal part of PrP^res^ was resistant to stringent conditions of proteolysis in brain material derived from the scrapie-infected macaque, as revealed with SAF-32 anti-PrP antibody: this is in contrast to PrP^res^ derived from c-BSE or L-type BSE-infected animals. We did not detect accumulation of PrP^res^ in peripheral organs (lymph nodes, spleen, tonsils) of this animal exposed through the intracerebral route.

At the time of this writing, the macaque exposed to the atypical (Nor98) scrapie isolate remains asymptomatic (>7 years post-inoculation). Similarly, we have not observed clinical signs in three macaques exposed to CWD isolates derived from naturally infected white-tailed deer or experimentally infected cattle (7 years post-inoculation). Amongst bovine prion strains, classical BSE (intracerebrally inoculated) induced prion disease was observed in 17 macaques after incubation periods ranging from about three to less than 8 years depending on the dose inoculated (100 mg to 50 μg), whereas 2.5 to 25 mg of brain tissue of L-type BSE intracerebrally inoculated into macaques resulted in an incubation period of 2 years. More than 10 years post-inoculation, an H-type BSE-exposed macaque also remains without neurological signs (Table 1).

## Discussion

We describe the transmission of spongiform encephalopathy in a non-human primate inoculated 10 years earlier with a strain of sheep c-scrapie. Because of this extended incubation period in a facility in which other prion diseases are under study, we are obliged to consider two alternative possibilities that might explain its occurrence. We first considered the possibility of a sporadic origin (like CJD in humans). Such an event is extremely improbable because the inoculated animal was 14 years old when the clinical signs appeared, i.e. about 40% through the expected natural lifetime of this species, compared to a peak age incidence of 60–65 years in human sporadic CJD, or about 80% through their expected lifetimes. Moreover, sporadic disease has never been observed in breeding colonies or primate research laboratories, most notably among hundreds of animals over several decades of study at the National Institutes of Health^25^, and in nearly twenty older animals continuously housed in our own facility.

The second possibility is a laboratory cross-contamination. Three facts make this possibility equally unlikely. First, handling of specimens in our laboratory is performed with fastidious attention to the avoidance of any such cross-contamination. Second, no laboratory cross-contamination has ever been documented in other primate laboratories, including the NIH, even between infected and uninfected animals housed in the same or adjacent cages with daily intimate contact (P. Brown, personal communication). Third, the cerebral lesion profile is different from all the other prion diseases we have studied in this model^19^, with a correlation between cerebellar lesions (massive spongiform change of Purkinje cells, intense PrP^res^ staining and reactive gliosis^26^) and ataxia. The iron deposits present in the globus pallidus are a non specific finding that have been reported previously in neurodegenerative diseases and aging^27^. Conversely, the thalamic lesion was reminiscent of a metabolic disease due to thiamine deficiency^28^ but blood thiamine levels were within normal limits (data not shown). The preferential distribution of spongiform change in cortex associated with a limited distribution in the brainstem is reminiscent of the lesion profile in MM2c and VV1 sCJD patients^29^, but interspecies comparison of lesion profiles should be interpreted with caution. It is of note that the same classical scrapie isolate induced TSE in C57Bl/6 mice with similar incubation periods and lesional profiles as a sample derived from a MM1 sCJD patient^30^.

We are therefore confident that the illness in this cynomolgus macaque represents a true transmission of a sheep c-scrapie isolate directly to an old-world monkey, which taxonomically resides in the primate subdivision (parvorder of catarrhini) that includes humans. With an homology of its PrP protein with humans of 96.4%^31^, cynomolgus macaque constitutes a highly relevant model for assessing zoonotic risk of prion diseases. Since our initial aim was to show the absence of transmission of scrapie to macaques in the worst-case scenario, we obtained materials from a flock of naturally-infected sheep, affecting animals with different genotypes^32^. This c-scrapie isolate exhibited complete transmission in ARQ/ARQ sheep (332 ± 56 days) and Tg338 transgenic mice expressing ovine VRQ/VRQ prion protein (220 ± 5 days) (O. Andreoletti, personal communication). From the standpoint of zoonotic risk, it is important to note that sheep with c-scrapie (including the isolate used in our study) have demonstrable infectivity throughout their lymphoreticular system early in the incubation period of the disease (3 months-old for all the lymphoid organs, and as early as 2 months-old in gut-associated lymph nodes)^33^. In addition, scrapie infectivity has been identified in blood^34^, milk^35^ and skeletal muscle^36^ from asymptomatic but scrapie infected small ruminants which implies a potential dietary exposure for consumers.

Two earlier studies have reported the occurrence of clinical TSE in cynomolgus macaques after exposures to scrapie isolates. In the first study, the “Compton” scrapie isolate (derived from an English sheep) and serially propagated for 9 passages in goats did not transmit TSE in cynomolgus macaque, rhesus macaque or chimpanzee within 7 years following intracerebral challenge^1^; conversely, after 8 supplementary passages in conventional mice, this “Compton” isolate induced TSE in a cynomolgus macaque 5 years after intracerebral challenge, but rhesus macaques and chimpanzee remained asymptomatic 8.5 years post-exposure^8^. However, multiple successive passages that are classically used to select laboratory-adapted prion strains can significantly modify the initial properties of a scrapie isolate, thus questioning the relevance of zoonotic potential for the initial sheep-derived isolate. The same isolate had also induced disease into squirrel monkeys (new-world monkey)^9^. A second historical observation reported that a cynomolgus macaque developed TSE 6 years post-inoculation with brain homogenate from a scrapie-infected Suffolk ewe (derived from USA), whereas a rhesus macaque and a chimpanzee exposed to the same inoculum remained healthy 9 years post-exposure^1^. This inoculum also induced TSE in squirrel monkeys after 4 passages in mice. Other scrapie transmission attempts in macaque failed but had more shorter periods of observation in comparison to the current study. Further, it is possible that there are differences in the zoonotic potential of different scrapie strains.

The most striking observation in our study is the extended incubation period of scrapie in the macaque model, which has several implications. Firstly, our observations constitute experimental evidence in favor of the zoonotic potential of c-scrapie, at least for this isolate that has been extensively studied^32^,^33^,^34^,^35^,^36^. The cross-species zoonotic ability of this isolate should be confirmed by performing duplicate intracerebral exposures and assessing the transmissibility by the oral route (a successful transmission of prion strains through the intracerebral route may not necessarily indicate the potential for oral transmission^37^). However, such confirmatory experiments may require more than one decade, which is hardly compatible with current general management and support of scientific projects; thus this study should be rather considered as a case report.

Secondly, transmission of c-BSE to primates occurred within 8 years post exposure for the lowest doses able to transmit the disease (the survival period after inoculation is inversely proportional to the initial amount of infectious inoculum). The occurrence of scrapie 10 years after exposure to a high dose (25 mg) of scrapie-infected sheep brain suggests that the macaque has a higher species barrier for sheep c-scrapie than c-BSE, although it is notable that previous studies based on *in vitro* conversion of PrP suggested that BSE and scrapie prions would have a similar conversion potential for human PrP^38^.

Thirdly, prion diseases typically have longer incubation periods after oral exposure than after intracerebral inoculations: since humans can develop Kuru 47 years after oral exposure^39^, an incubation time of several decades after oral exposure to scrapie would therefore be expected, leading the disease to occur in older adults, i.e. the peak age for cases considered to be sporadic disease, and making a distinction between scrapie-associated and truly sporadic disease extremely difficult to appreciate.

Fourthly, epidemiologic evidence is necessary to confirm the zoonotic potential of an animal disease suggested by experimental studies. A relatively short incubation period and a peculiar epidemiological situation (e.g., all the first vCJD cases occurring in the country with the most important ongoing c-BSE epizootic) led to a high degree of suspicion that c-BSE was the cause of vCJD. Sporadic CJD are considered spontaneous diseases with an almost stable and constant worldwide prevalence (0.5–2 cases per million inhabitants per year), and previous epidemiological studies were unable to draw a link between sCJD and classical scrapie^6^,^7^,^40^,^41^, even though external causes were hypothesized to explain the occurrence of some sCJD clusters^42^,^43^,^44^. However, extended incubation periods exceeding several decades would impair the predictive values of epidemiological surveillance for prion diseases, already weakened by a limited prevalence of prion diseases and the multiplicity of isolates gathered under the phenotypes of “scrapie” and “sporadic CJD”.

Fifthly, considering this 10 year-long incubation period, together with both laboratory and epidemiological evidence of decade or longer intervals between infection and clinical onset of disease, no premature conclusions should be drawn from negative transmission studies in cynomolgus macaques with less than a decade of observation, as in the aforementioned historical transmission studies of scrapie to primates^1^,^8^,^9^. Our observations and those of others^45^,^46^ to date are unable to provide definitive evidence regarding the zoonotic potential of CWD, atypical/Nor98 scrapie or H-type BSE. The extended incubation period of the scrapie-affected macaque in the current study also underscores the limitations of rodent models expressing human PrP for assessing the zoonotic potential of some prion diseases since their lifespan remains limited to approximately two years^21^,^47^,^48^. This point is illustrated by the fact that the recently reported transmission of scrapie to humanized mice was not associated with clinical signs for up to 750 days and occurred in an extreme minority of mice with only a marginal increase in attack rate upon second passage^13^. The low attack rate in these studies is certainly linked to the limited lifespan of mice compared to the very long periods of observation necessary to demonstrate the development of scrapie. Alternatively, one could estimate that a successful second passage is the result of strain adaptation to the species barrier, thus poorly relevant of the real zoonotic potential of the original scrapie isolate of sheep origin^49^. The development of scrapie in this primate after an incubation period compatible with its lifespan complements the study conducted in transgenic (humanized) mice; taken together these studies suggest that some isolates of sheep scrapie can promote misfolding of the human prion protein and that scrapie can develop within the lifespan of some primate species.

In addition to previous studies on scrapie transmission to primate^1^,^8^,^9^ and the recently published study on transgenic humanized mice^13^, our results constitute new evidence for recommending that the potential risk of scrapie for human health should not be dismissed. Indeed, human PrP transgenic mice and primates are the most relevant models for investigating the human transmission barrier. To what extent such models are informative for measuring the zoonotic potential of an animal TSE under field exposure conditions is unknown. During the past decades, many protective measures have been successfully implemented to protect cattle from the spread of c-BSE, and some of these measures have been extended to sheep and goats to protect from scrapie according to the principle of precaution. Since cases of c-BSE have greatly reduced in number, those protective measures are currently being challenged and relaxed in the absence of other known zoonotic animal prion disease. We recommend that risk managers should be aware of the long term potential risk to human health of at least certain scrapie isolates, notably for lymphotropic strains like the classical scrapie strain used in the current study. Relatively high amounts of infectivity in peripheral lymphoid organs in animals infected with these strains could lead to contamination of food products produced for human consumption. Efforts should also be maintained to further assess the zoonotic potential of other animal prion strains in long-term studies, notably lymphotropic strains with high prevalence like CWD, which is spreading across North America, and atypical/Nor98 scrapie (Nor98)^50^ that was first detected in the past two decades and now represents approximately half of all reported cases of prion diseases in small ruminants worldwide, including territories previously considered as scrapie free. Even if the prevailing view is that sporadic CJD is due to the spontaneous formation of CJD prions, it remains possible that its apparent sporadic nature may, at least in part, result from our limited capacity to identify an environmental origin.

## Materials and Methods

### Ethics statement

Primates were housed and handled in accordance with the European Directive 2010/63 related to animal protection and welfare in research, under constant internal surveillance of veterinarians. Animals were handled under anesthesia to limit stress, and euthanasia was performed for ethical reasons when animals lost autonomy. All the experimental protocols were approved by the French Atomic Energy Commission Ethical Committee (CETEA, approval 12-047).

### Experimental animals

Captive-bred 2–5 year-old male cynomolgus macaques (*Macaca fascicularis*) were provided by Noveprim (Mauritius), checked for the absence of common primate pathogens before importation, and handled in accordance to national guidelines. They were all homozygous for methionine at *PRNP* codon 129. Life expectancy of cynomolgus macaques in captivity is 35 to 39 years. Animals housed in level-3 animal care facilities (agreement numbers A 92-032-02 for animal care facilities, 92-189 for animal experimentation) were daily controlled by animal caretaker and regularly examined at least once a week by veterinarians.

### Experimental inoculations

The classical scrapie inoculum was derived from the cerebellum of a French Romanov sheep (ARQ/ARQ homozygote at codons 136, 154 and 171 for PrP gene) naturally infected with classical scrapie (21 kDa PrPres), and the atypical scrapie (Nor98) inoculum was derived from the cerebellum of a Norwegian sheep naturally infected with Nor98 (ARQ/ARR). The cattle-derived inocula were issued from a pool of 11 naturally infected UK cattle (c-BSE)^51^, an Italian natural case (L-type BSE, case #1088^52^) or a French natural case (H-type BSE, case #A1F^53^). The CWD-related inocula were derived from either experimentally infected cattle (case #599 infected with mule deer (MD)-derived CWD or case #611 infected with white-tailed deer (WTD)-derived CWD^54^,^55^), or directly from infected white-tailed deer (case #654 from the same experiments).

These samples were homogenized (10% weight/volume) in a 5% glucose solution, and the equivalent of 2.5 to 100 mg of brain inocula were intracerebrally (i.c.) inoculated to macaques under anesthesia. When specified, the equivalent of 8 mg of brain inocula was injected into tonsils (intratonsilar, i.to) to model a peripheral exposure. Inoculation were performed according procedures established to avoid any cross-contamination (inoculation of unique inoculum at a single time, use of disposable materials, systematic double checking).

### Neuropathology and immunohistochemistry

Tissues were fixed in 10% formalin for processing for microscopic examination. Neuropathological methods and immunohistochemical detection of pathological prion protein (PrP^sc^) were performed on brain sections as previously described^56^. Briefly, PrP was detected using the Discovery Automated IHC Stainer. After dewaxing with Ventana EZ Prep solution, slides were sequentially incubated with the cell conditioner 1 protocol (CC1) for 48 minutes, guanidinium thiocyanate 4.0 M for 16 minutes, the primary mouse monoclonal antibody (3F4 or 12F10) for 40 minutes, the secondary anti-mouse antibody OmniMap anti-Ms HRP (Ventana) for 16 minutes. Reactions were developed with the ChromoMap DAB kit (Ventana).

### PrP^res^ analysis

PrP^**res**^ was purified according to the TeSeE purification protocol (Bio-Rad), in adapted conditions of proteolysis for strain discrimination as previously described^19^, using Sha-31 or SAF-32 antibodies.

## Additional Information

**How to cite this article**: Comoy, E. E. *et al*. Transmission of scrapie prions to primate after an extended silent incubation period. *Sci. Rep*. **5**, 11573; doi: 10.1038/srep11573 (2015).

## Supplementary Material

Supplementary Information

## Figures and Tables

**Table 1:**
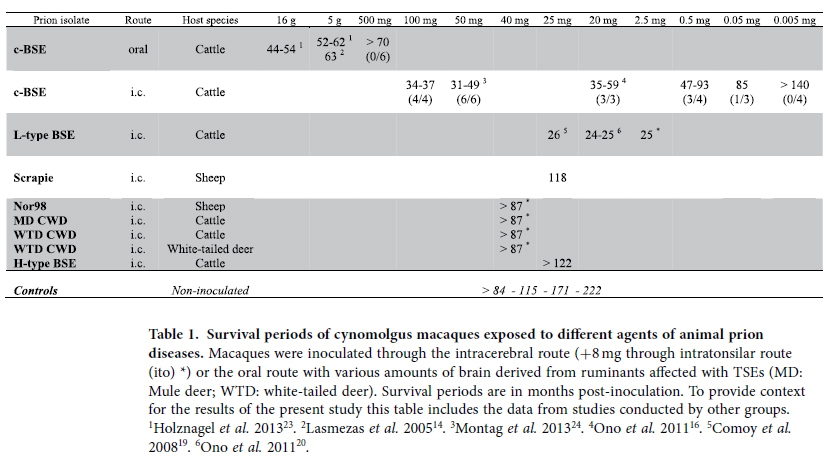
Survival periods of cynomolgus macaques exposed to different agents of animal prion diseases.

## References

[b1] Gajdusek D. C. Unconventional viruses and the origin and disappearance of kuru. Science 197, 943–960 (1977). 142303 10.1126/science.142303

[b2] Brown P. . Iatrogenic Creutzfeldt-Jakob disease, final assessment. Emerg Infect Dis 18, 901–907, 10.3201/eid1806.120116 (2012). 22607808 PMC3358170

[b3] Ironside J. W. . A new variant of Creutzfeldt-Jakob disease: neuropathological and clinical features. Cold Spring Harb Symp Quant Biol 61, 523–530 (1996). 9246478

[b4] Lasmezas C. I. . BSE transmission to macaques. Nature 381, 743–744, 10.1038/381743a0 (1996). 8657276

[b5] Bruce M. E. . Transmissions to mice indicate that ‘new variant’ CJD is caused by the BSE agent. Nature 389, 498–501, 10.1038/39057 (1997). 9333239

[b6] Chatelain J. . Epidemiologic comparisons between Creutzfeldt-Jakob disease and scrapie in France during the 12-year period 1968-1979. J Neurol Sci 51, 329–337 (1981). 7024478 10.1016/0022-510x(81)90111-8

[b7] Masters C. L. . Creutzfeldt-Jakob disease: patterns of worldwide occurrence and the significance of familial and sporadic clustering. Ann Neurol 5, 177–188, 10.1002/ana.410050212 (1979). 371520

[b8] Gibbs C. J. Jr. & Gajdusek D. C. Transmission of scrapie to the cynomolgus monkey (Macaca fascicularis). Nature 236, 73–74 (1972). 4623139 10.1038/236073a0

[b9] Gibbs C. J. Jr. & Gajdusek D. C. Experimental subacute spongiform virus encephalopathies in primates and other laboratory animals. Science 182, 67–68 (1973). 4199733 10.1126/science.182.4107.67

[b10] Baker H. F., Ridley R. M. & Wells G. A. Experimental transmission of BSE and scrapie to the common marmoset. The Veterinary record 132, 403–406 (1993). 8488658 10.1136/vr.132.16.403

[b11] BIOHAZ E. Joint scientific opinion on any possible epidemiological or molecular association between TSEs in animals and humans. EFSA J. 9, 111 (2011).

[b12] Hadlow W. J., Prusiner S. B., Kennedy R. C. & Race R. E. Brain tissue from persons dying of Creutzfeldt-Jakob disease causes scrapie-like encephalopathy in goats. Ann Neurol 8, 628–632, 10.1002/ana.410080615 (1980). 7011169

[b13] Cassard H. . Evidence for zoonotic potential of ovine scrapie prions. Nat Commun 5, 5821–5830, 10.1038/ncomms6821 (2014). 25510416

[b14] Lasmezas C. I. . Risk of oral infection with bovine spongiform encephalopathy agent in primates. Lancet 365, 781–783, 10.1016/S0140-6736(05)17985-9 (2005). 15733719

[b15] Herzog C. . PrPTSE distribution in a primate model of variant, sporadic, and iatrogenic Creutzfeldt-Jakob disease. Journal of virology 79, 14339–14345, 10.1128/JVI.79.22.14339-14345.2005 (2005). 16254368 PMC1280201

[b16] Ono F. . Experimental transmission of bovine spongiform encephalopathy (BSE) to cynomolgus macaques, a non-human primate. Jpn J Infect Dis 64, 50–54 (2011). 21266755

[b17] Chen C. C., Wang Y. H. & Wu K. Y. Consumption of bovine spongiform encephalopathy (BSE) contaminated beef and the risk of variant Creutzfeldt-Jakob disease. Risk Anal 33, 1958–1968, 10.1111/risa.12079 (2013). 23755826

[b18] Chen C. C. & Wang Y. H. Estimation of the Exposure of the UK Population to the Bovine Spongiform Encephalopathy Agent through Dietary Intake During the Period 1980 to 1996. PLoS One 9, e94020, 10.1371/journal.pone.0094020 (2014). 24736322 PMC3988046

[b19] Comoy E. E. . Atypical BSE (BASE) transmitted from asymptomatic aging cattle to a primate. PLoS One 3, e3017, 10.1371/journal.pone.0003017 (2008). 18714385 PMC2515088

[b20] Ono F. . Atypical L-type bovine spongiform encephalopathy (L-BSE) transmission to cynomolgus macaques, a non-human primate. Jpn J Infect Dis 64, 81–84 (2011). 21266763

[b21] Beringue V. . Transmission of atypical bovine prions to mice transgenic for human prion protein. Emerg Infect Dis 14, 1898–1901, 10.3201/eid1412.080941 (2008). 19046515 PMC2634647

[b22] Kong Q. . Evaluation of the human transmission risk of an atypical bovine spongiform encephalopathy prion strain. Journal of virology 82, 3697–3701, 10.1128/JVI.02561-07 (2008). 18234793 PMC2268471

[b23] Holznagel E. . Foodborne transmission of bovine spongiform encephalopathy to nonhuman primates. Emerg Infect Dis 19, 712–720, 10.3201/eid1905.120274 (2013). 23647575 PMC3647490

[b24] Montag J., Schulz-Schaeffer W., Schrod A., Hunsmann G. & Motzkus D. Asynchronous onset of clinical disease in BSE-infected macaques. Emerg Infect Dis 19, 1125–1127, 10.3201/eid1907.120438 (2013). 23764183 PMC3713963

[b25] Brown P. . Human spongiform encephalopathy: the National Institutes of Health series of 300 cases of experimentally transmitted disease. Ann Neurol 35, 513–529, 10.1002/ana.410350504 (1994). 8179297

[b26] Dearmond S. J., Kretzschmar H. & Prusiner S. B. in Greenfield’s Neuropathology, 7th Edition Vol. 2 (eds Graham DI & Lantos D ) Ch. 5, 49 (Arnold, 2002).

[b27] Hallgren B. & Sourander P. The effect of age on the non-haemin iron in the human brain. J Neurochem 3, 41–51 (1958). 13611557 10.1111/j.1471-4159.1958.tb12607.x

[b28] Harper C. & Butterworth R. in Greenfield’s Neuropathology 7th Edition Vol. 1 (eds Graham DI & Lantos D ) Ch. 10, 610–652 (Arnold, 2002).

[b29] Parchi P. . Classification of sporadic Creutzfeldt-Jakob disease based on molecular and phenotypic analysis of 300 subjects. Ann Neurol 46, 224–233 (1999). 10443888

[b30] Lasmezas C. I. . Adaptation of the bovine spongiform encephalopathy agent to primates and comparison with Creutzfeldt– Jakob disease: implications for human health. Proc Natl Acad Sci U S A 98, 4142–4147, 10.1073/pnas.041490898 (2001). 11259641 PMC31193

[b31] Schatzl H. M., Da Costa M., Taylor L., Cohen F. E. & Prusiner S. B. Prion protein gene variation among primates. J Mol Biol 245, 362–374 (1995). 7837269 10.1006/jmbi.1994.0030

[b32] Touzeau S. . Modelling the spread of scrapie in a sheep flock: evidence for increased transmission during lambing seasons. Arch Virol 151, 735–751, 10.1007/s00705-005-0666-y (2006). 16307175

[b33] Andreoletti O. . Early accumulation of PrP(Sc) in gut-associated lymphoid and nervous tissues of susceptible sheep from a Romanov flock with natural scrapie. J Gen Virol 81, 3115–3126 (2000). 11086143 10.1099/0022-1317-81-12-3115

[b34] Lacroux C. . Prionemia and leukocyte-platelet-associated infectivity in sheep transmissible spongiform encephalopathy models. Journal of virology 86, 2056–2066, 10.1128/JVI.06532-11 (2012). 22156536 PMC3302392

[b35] Lacroux C. . Prions in milk from ewes incubating natural scrapie. PLoS Pathog 4, e1000238, 10.1371/journal.ppat.1000238 (2008). 19079578 PMC2587713

[b36] Andreoletti O. . PrPSc accumulation in myocytes from sheep incubating natural scrapie. Nat Med 10, 591–593, 10.1038/nm1055 (2004). 15156203

[b37] Wells G. A. . Studies of the transmissibility of the agent of bovine spongiform encephalopathy to pigs. J Gen Virol 84, 1021–1031 (2003). 12655106 10.1099/vir.0.18788-0

[b38] Raymond G. J. . Molecular assessment of the potential transmissibilities of BSE and scrapie to humans. Nature 388, 285–288, 10.1038/40876 (1997). 9230438

[b39] Collinge J. . Kuru in the 21st century–an acquired human prion disease with very long incubation periods. Lancet 367, 2068–2074, 10.1016/S0140-6736(06)68930-7 (2006). 16798390

[b40] van Duijn C. M. . Case-control study of risk factors of Creutzfeldt-Jakob disease in Europe during 1993–95. European Union (EU) Collaborative Study Group of Creutzfeldt-Jakob disease (CJD). Lancet 351, 1081–1085 (1998). 9660576 10.1016/s0140-6736(97)09468-3

[b41] Papacostas S., Malikides A., Petsa M. & Kyriakides T. Ten-year mortality from Creutzfeldt-Jakob disease in Cyprus. East Mediterr Health J 14, 715–719 (2008). 18720636

[b42] Linsell L. . A case-control study of sporadic Creutzfeldt-Jakob disease in the United Kingdom: analysis of clustering. Neurology 63, 2077–2083 (2004). 15596753 10.1212/01.wnl.0000145844.53251.bc

[b43] Doi Y. . Spatial clusters of Creutzfeldt-Jakob disease mortality in Japan between 1995 and 2004. Neuroepidemiology 30, 222–228, 10.1159/000126916 (2008). 18424903 PMC2790766

[b44] Moreno M. J. . Creutzfeldt-Jakob disease cluster in the health area of Meixoeiro Hospital. Acta Neurol Scand 127, 38–45, 10.1111/j.1600-0404.2012.01678.x (2013). 22590993

[b45] Race B. . Susceptibilities of nonhuman primates to chronic wasting disease. Emerg Infect Dis 15, 1366–1376, 10.3201/eid1509.090253 (2009). 19788803 PMC2819871

[b46] Race B. . Chronic wasting disease agents in nonhuman primates. Emerg Infect Dis 20, 833–837, 10.3201/eid2005.130778 (2014). 24751215 PMC4012792

[b47] Wadsworth J. D. . Atypical scrapie prions from sheep and lack of disease in transgenic mice overexpressing human prion protein. Emerg Infect Dis 19, 1731–1739, 10.3201/eid1911.121341 (2013). 24188521 PMC3837652

[b48] Wilson R. . Chronic wasting disease and atypical forms of bovine spongiform encephalopathy and scrapie are not transmissible to mice expressing wild-type levels of human prion protein. J Gen Virol 93, 1624–1629, 10.1099/vir.0.042507-0 (2012). 22495232

[b49] Dickinson A. G., Fraser H. & Outram G. W. Scrapie incubation time can exceed natural lifespan. Nature 256, 732–733 (1975). 807857 10.1038/256732a0

[b50] Benestad S. L. . Cases of scrapie with unusual features in Norway and designation of a new type, Nor98. The Veterinary record 153, 202–208 (2003). 12956297 10.1136/vr.153.7.202

[b51] Yutzy B. . Time-course studies of 14-3-3 protein isoforms in cerebrospinal fluid and brain of primates after oral or intracerebral infection with bovine spongiform encephalopathy agent. J Gen Virol 88, 3469–3478, 10.1099/vir.0.83128-0 (2007). 18024918

[b52] Casalone C. . Identification of a second bovine amyloidotic spongiform encephalopathy: molecular similarities with sporadic Creutzfeldt-Jakob disease. Proc Natl Acad Sci U S A 101, 3065–3070, 10.1073/pnas.0305777101 (2004). 14970340 PMC365745

[b53] Biacabe A. G., Laplanche J. L., Ryder S. & Baron T. Distinct molecular phenotypes in bovine prion diseases. EMBO Rep 5, 110–115, 10.1038/sj.embor.7400054 (2004). 14710195 PMC1298965

[b54] Hamir A. N., Kunkle R. A., Miller J. M., Greenlee J. J. & Richt J. A. Experimental second passage of chronic wasting disease (CWD(mule deer)) agent to cattle. J Comp Pathol 134, 63–69, 10.1016/j.jcpa.2005.07.001 (2006). 16423572

[b55] Hamir A. N., Miller J. M., Kunkle R. A., Hall S. M. & Richt J. A. Susceptibility of cattle to first-passage intracerebral inoculation with chronic wasting disease agent from white-tailed deer. Veterinary Pathology 44, 487–493, 10.1354/vp.44-4-487 (2007). 17606510

[b56] Lescoutra-Etchegaray N. . evaluation of the protection of primates transfused with vCJD-infected blood products filtered with prion removal devices: a five-year update. Transfusion, 10.1111/trf.12999 (2015). 25647476

