# Supplementary material for: Transmission of scrapie prions to primate after an extended silent incubation period
Source: Sci Rep. 2015 Jun 30;5:11573. doi: 10.1038/srep11573 (PMC4485159; doi:10.1038/srep11573)
Supplement: Supplementary Information [file srep11573-s1.pdf]

Transmission of scrapie prions to primate after an extended silent incubation period

Emmanuel E. Comoy, Jacqueline Mikol, Sophie Luccantoni-Freire, Evelyne Correia, Nathalie Lescoutra-Etchegaray, Valérie Durand, Capucine Dehen, Olivier Andreoletti, Cristina Casalone, Juergen A. Richt, Justin J. Greenlee, Thierry Baron, Sylvie L. Benestad, Paul Brown and Jean-Philippe Deslys

Supplementary figure 1: Comparison of PrP immunoreactivity between healthy and scrapie-infected primate

A) Absence of detection of PrP^{C} in the brain of a healthy primate (x3.6, 3F4 monoclonal antibody). B) Strong PrP^{Sc} immunoreactivity in the brain of scrapie-infected primate (x3.6, 3F4). It must be noted that the scrapie-infected primate brain is smaller due to atrophy.

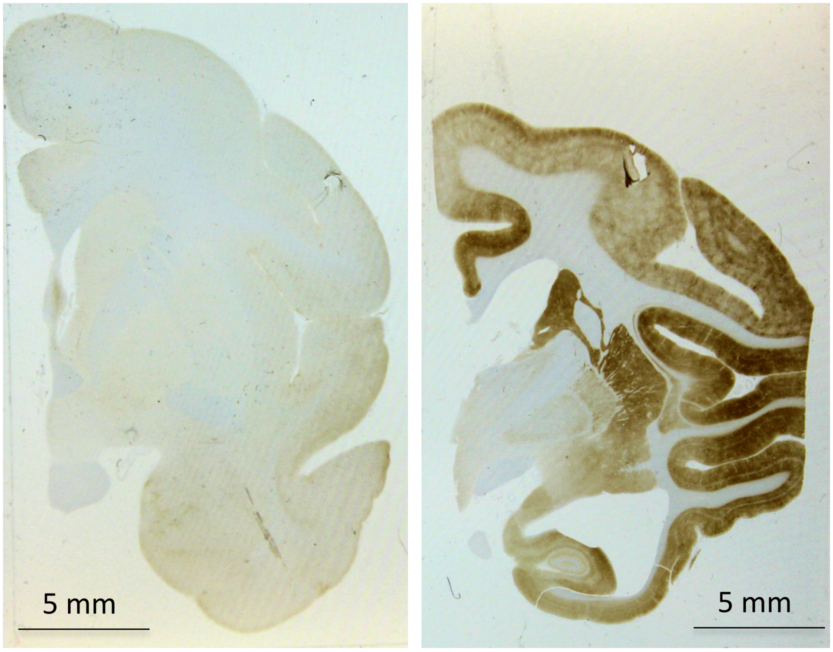
5 mm
5 mm
